# The weight of complications: high and low BMI have disparate modes of failure in total hip arthroplasty

**DOI:** 10.1186/s42836-024-00233-7

**Published:** 2024-03-04

**Authors:** Jessica Schmerler, Victoria E. Bergstein, William ElNemer, Andrew B. Harris, Harpal S. Khanuja, Uma Srikumaran, Vishal Hegde

**Affiliations:** Department of Orthopaedic Surgery, The Johns Hopkins University School of Medicine, 601 N Caroline St, Baltimore, MD 21287 USA

**Keywords:** Revision total hip arthroplasty, Total hip arthroplasty, Revision surgery, BMI

## Abstract

**Background:**

Body mass index (BMI) has been shown to influence risk for revision total hip arthroplasty (rTHA), but few studies have specifically examined which causes of rTHA are most likely in different BMI classes. We hypothesized that patients in different BMI classes would undergo rTHA for disparate reasons.

**Methods:**

Ninety-eight thousand six hundred seventy patients undergoing rTHA over 2006–2020 were identified in the National Inpatient Sample. Patients were classified as underweight, normal-weight, overweight/obese, or morbidly obese. Multivariable logistic regression was used to analyze the impact of BMI on rTHA for periprosthetic joint infection (PJI), dislocation, periprosthetic fracture (PPF), aseptic loosening, or mechanical complications. Analyses were adjusted for age, sex, race/ethnicity, socioeconomic status, insurance, geographic region, and comorbidities.

**Results:**

Compared to normal-weight patients, underweight patients were 131% more likely to have a revision due to dislocation and 63% more likely due to PPF. Overweight/obese patients were 19% less likely to have a revision due to dislocation and 10% more likely due to PJI. Cause for revision in morbidly obese patients was 4s1% less likely to be due to dislocation, 8% less likely due to mechanical complications, and 90% more likely due to PJI.

**Conclusions:**

Overweight/obese and morbidly obese patients were more likely to undergo rTHA for PJI and less likely for mechanical reasons compared to normal weight patients. Underweight patients were more likely to undergo rTHA for dislocation or PPF. Understanding the differences in cause for rTHA among the BMI classes can aid in patient-specific optimization and management to reduce postoperative complications.

**Level of evidence:**

III.

**Supplementary Information:**

The online version contains supplementary material available at 10.1186/s42836-024-00233-7.

## Background

Total hip arthroplasty (THA) is one of the most common orthopaedic surgeries worldwide, with over one million procedures performed annually [1]. THA is safe and effective, with a success rate of up to 90% at 10 years post-surgery and 80% after 25 years [2–4]. The most common causes of THA failures resulting in revision THA (rTHA) are aseptic loosening, followed by dislocation or instability, infection, mechanical complications, fracture, and pain [2, 4]. Patients undergoing rTHA often experience longer operative times, greater blood loss, and greater risk of complications compared to primary THA [5, 6]. Given that the annual volume of THA procedures is expected to nearly triple by 2040 [7], it is critical to understand the contributors to revision risk and the causes for revision in different patient populations. This will in turn allow for patient-specific perioperative risk mitigation to provide optimal care for patients undergoing THA.

One patient-level characteristic that has been extensively studied in the setting of THA outcomes is body mass index (BMI). Studies have demonstrated a clear association between obesity and lower extremity osteoarthritis (OA), which, in turn, results in the need for THA [5, 8–10]. Many studies have investigated the relationship between obesity and adverse events in THA, demonstrating that obese patients are more likely to experience postoperative complications, increased likelihood of revision, worse functional outcomes, and longer operative times than non-obese patients [11, 12]. This has led to many arthroplasty surgeons implementing BMI cutoffs of 35 kg/m^2^ or 40 kg/m^2^ for performing THA [13, 14]. On the other hand, albeit markedly less studied, underweight patients have been shown to experience increased risk for complications and revisions as well [15–17]. These data indicate the presence of a “J-shaped” curve with respect to complications and revisions over the BMI spectrum, with both underweight and obese patients at increased risk. However, despite substantial literature existing on complications and revision risk in underweight and obese patients, there is a lack of evidence detailing specifically which causes of revision are most likely in patients of different BMI classes.

The aim of the present study is therefore to characterize the differences in the likelihood of various causes of rTHA, stratified by BMI class both above and below the normal range. As underweight patients experience different comorbidities and biomechanical stress on their joints compared to overweight/obese patients, we hypothesize that patients of different BMI classes will be more likely to undergo rTHA for different indications. Beyond merely understanding the most common causes for revision across all patients, understanding BMI-specific differences may allow for patient-specific optimization, education, and changes in surgical technique for patients of different BMI classes.

## Methods

This study was exempt from institutional board review approval.

### Data source and study population

Patients greater than 18 years old who underwent rTHA over 2006–2020 were identified in the National Inpatient Sample (NIS), maintained by the Healthcare Cost and Utilization Project. The International Classification of Diseases, Ninth Revision (ICD-9), and Tenth Revision (ICD-10) procedure codes (detailed in Table S1, Supplementary Information) were used for rTHA. Patients were then stratified into four BMI classes: underweight (BMI < 19 kg/m^2^), normal-weight, overweight/obese (BMI 25–39.9 kg/m^2^), and morbidly obese (BMI ≥ 40 kg/m^2^), with patients above normal-weight stratified as such based on traditionally-used BMI cutoffs of 40 kg/m^2^ [18]. BMI classes were identified with the ICD diagnosis codes detailed in Supplemental Table S1 [19]. ICD codes used in the NIS refer to those in the patient’s chart during the hospitalization, thus it was assumed that these were accurate indicators of a patient’s BMI class at the time of surgery.

Of the 98,670 patients who underwent rTHA over the period analyzed, 351 (0.4%) were underweight, 82,246 (83.4%) were normal-weight, 9,510 (9.6%) were overweight/obese, and 6,563 (6.7%) were morbidly obese. Table 1 presents the characteristics of patients in each BMI class undergoing rTHA. The patient population was 56.0% women and was on average 66.5 ± 12.8 years old with an average Elixhauser Comorbidity Index (ECI) score of 2.2 ± 1.8. Age, sex, race/ethnicity, socioeconomic status, payer status, hospital setting, and ECI all varied significantly by BMI class (*P* < 0.001 for all).

**Table 1:** Characteristics of patients undergoing revision THA by BMI class

**Parameter**	**Total sample**	**Underweight**	**Normal weight**	**Overweight/ obese**	**Morbidly obese**	***P*-value**
N (%)	98,670	351 (0.4%)	82,246 (83.4%)	9,510 (9.6%)	6,563 (6.7%)	
Age (SD)	66.5 (12.8)	72.3 (13.8)	67.2 (13.0)	63.8 (11.1)	61.7 (10.3)	< 0.001
Sex						< 0.001
Men	43,401 (44.0%)	98 (27.9%)	36,729 (44.7%)	4,219 (44.4%)	2,355 (35.9%)	
Women	55,269 (56.0%)	253 (72.1%)	45,517 (55.3%)	5,291 (55.6%)	4,208 (64.1%)	
Race/Ethnicity						< 0.001
White	85,413 (86.6%)	309 (88.0%)	71,685 (87.2%)	8,011 (84.2%)	5,408 (82.4%)	
Black	8.170 (8.3%)	27 (7.7%)	6,335 (7.7%)	944 (9.9%)	864 (13.2%)	
Hispanic	4,238 (4.3%)	*	3,477 (4.2%)	494 (5.2%)	261 (4.0%)	
Asian American/ Pacific Islander	849 (0.9%)	*	749 (0.9%)	61 (0.6%)	30 (0.5%)	
Income Quartile						< 0.001
Q1	22,699 (23.0%)	104 (29.6%)	18,672 (22.7%)	2,166 (22.8%)	1,757 (26.8%)	
Q2	25,537 (25.9%)	98 (27.9%)	21,136 (25.7%)	2,492 (26.2%)	1,811 (27.6%)	
Q3	25,248 (25.6%)	71 (20.2%)	21,050 (25.6%)	2,438 (25.6%)	1,689 (25.7%)	
Q4	25,186 (25.5%)	78 (22.2%)	21,388 (26.0%)	2,414 (25.4%)	1,306 (19.9%)	
Payer Status						< 0.001
Medicare	61,709 (62.5%)	275 (78.4%)	52,521 (63.9%)	5,300 (55.7%)	3,613 (55.1%)	
Medicaid	4,550 (4.6%)	12 (3.4%)	3,687 (4.5%)	463 (4.9%)	388 (5.9%)	
Private	28,281 (28.7%)	58 (16.5%)	22,648 (27.5%)	3,273 (34.4%)	2,302 (35.1%)	
Self-Pay	750 (0.8%)	*	635 (0.8%)	66 (0.7%)	47 (0.7%)	
No Charge	135 (0.1%)	*	111 (0.1%)	14 (0.2%)	*	
Other	3,245 (3.3%)	*	2,644 (3.2%)	394 (4.1%)	203 (3.1%)	
Hospital Setting						< 0.001
Rural	6,456 (6.5%)	24 (6.8%)	5,449 (6.6%)	558 (5.9%)	425 (6.5%)	
Urban Non-Teaching	34,693 (35.2%)	118 (33.6%)	29,394 (35.7%)	3,015 (31.7%)	2,166 (33.0%)	
Urban Teaching	57,521 (58.3%)	209 (59.5%)	47,403 (57.6%)	5,937 (62.4%)	3,972 (60.5%)	
Elixhauser Comorbidity Index Score (SD)	2.2 (1.8)	3.3 (2.2)	2.0 (1.7)	3.1 (1.9)	3.5 (2.0)	< 0.001

^*^Cells with values ≤ 10 and their corresponding rows are not reported in order to avoid deidentification of NIS data

### Variables of interest

#### Outcomes of interest

The primary outcome was the likelihood of rTHA being performed for one of several indications. Indications for revision were grouped into eight categories (aseptic loosening, dislocation, mechanical complications, periprosthetic/total joint fracture, osteolysis/polyethylene wear, periprosthetic joint infection, arthrofibrosis, and other complications) using the ICD codes detailed in Table S1 [20].

#### Covariates

Covariates included age, sex (men/women), race/ethnicity (White, Black, Hispanic, or Asian American/Pacific Islander), payer status (Medicare, Medicaid, private insurance, or self-pay), socioeconomic status, and hospital setting (rural, urban non-teaching, or urban teaching). For socioeconomic status, the quartile classification of the estimated median household income of residents in the patient’s zip code (Table S2) was used as a proxy [21], with patients in Q1 residing in zip codes of low median income and patients in Q4 residing in zip codes of high median income. Also included as a covariate was the ECI, identified using The Elixhauser Stata package, which uses 31 patient comorbidities to calculate the ECI. The ECI was developed based on a study on the impact of a comprehensive set of comorbidities and their impact on commonly reported outcomes, such as length of stay, mortality, and in-hospital charges [22]. Comorbidities in the index include hypertension, heart failure, diabetes, anemia, substance use disorders and other psychiatric conditions, cancer, and liver disease, among others.

### Statistical analysis

Univariate chi-square analysis was used to analyze differences in indication for revision among BMI classes. Multivariable logistic regression models controlling for age, sex, race/ethnicity, payer status, hospital setting, and ECI were then constructed to examine the effect of BMI on likelihood of rTHA being due to each different indication for revision. The reference group for the models was normal-weight patients, which lacked ICD-9 codes for any BMI class.

Data were analyzed using Stata Statistical Software: Release 17; 2021 (StataCorp; College Station, TX, USA). A *P* value of < 0.05 was considered statistically significant.

## Results

Indication for revision varied significantly by BMI class (*P* < 0.001). The full results of univariate outcome modeling are presented in Table 2, and the full results of multivariable outcome modeling are presented in Table 3.

**Table 2:** Cause for revision THA stratified by BMI class

Cause for revision	Total sample	Underweight	Normal weight	Overweight/ obese	Morbidly obese	*P*-value*
Aseptic Loosening	13,923 (14.1%)	26 (7.4%)	11,992 (14.6%)	1,189 (12.5%)	716 (10.9%)	< 0.001
Dislocation	15,051 (15.3%)	109 (31.1%)	13,214 (16.1%)	1,153 (12.1%)	575 (8.8%)	< 0.001
Mechanical Complication	17,918 (18.2%)	35 (10.0%)	15,317 (18.6%)	1,619 (17.0%)	947 (14.4%)	< 0.001
Periprosthetic/ Total Joint Fracture	3,817 (3.9%)	28 (8.0%)	3,288 (4.0%)	292 (3.1%)	209 (3.2%)	< 0.001
Osteolysis/ Polyethylene Wear	5,270 (5.3%)	11 (3.1%)	4,568 (5.6%)	473 (5.0%)	218 (3.3%)	< 0.001
Periprosthetic Joint Infection	30,469 (30.9%)	98 (27.9%)	23,579 (28.7%)	3,423 (36.0%)	3,369 (51.3%)	< 0.001
Arthrofibrosis	49 (0.1%)	**	40 (0.1%)	**	**	0.91
Other Joint Complication	12,221 (12.4%)	44 (12.5%)	10,290 (12.5%)	1,357 (14.3%)	530 (8.1%)	< 0.001

^*^The *P*-value refers to that of the overall univariate analysis, thus, a *P*-value of < 0.05 indicates that the variable investigated in that row statistically varies significantly by weight class

^**^Cells with values ≤ 10 and their corresponding rows are not reported in order to avoid deidentification of NIS data

**Table 3:** Risk for each cause for revision by BMI class relative to normal weight

	**Underweight**			**Overweight/obese**			**Morbidly obese**		
Cause for Revision	OR	95% CI	*P*-value	OR	95% CI	*P*-value	OR	95% CI	*P*-value
Aseptic Loosening	**0.56**	**0.38–0.84**	**0.01**	1.02	0.95–1.08	0.65	0.93	0.86–1.01	0.09
Dislocation	**2.31**	**1.84–2.91**	** < 0.001**	**0.81**	**0.76–0.87**	** < 0.001**	**0.59**	**0.54–0.65**	** < 0.001**
Mechanical Complication	**0.63**	**0.44–0.89**	**0.01**	1.05	0.99–1.11	0.13	**0.92**	**0.85–0.99**	**0.02**
Periprosthetic/ Total Joint Fracture	**1.63**	**1.10–2.41**	**0.02**	0.90	0.79–1.02	0.10	1.04	0.90–1.21	0.57
Osteolysis/ Polyethylene Wear	0.77	0.42–1.40	0.39	**1.12**	**1.01–1.24**	**0.03**	**0.81**	**0.70–0.93**	**0.003**
Periprosthetic Joint Infection	0.84	0.66–1.07	0.16	**1.10**	**1.05–1.15**	** < 0.001**	**1.90**	**1.80–2.00**	** < 0.001**
Arthrofibrosis	-	-	-	1.58	0.65–3.85	0.31	1.17	0.35–3.94	0.80
Other Joint Complication	0.66	0.47–0.93	0.02	0.95	0.89–1.91	0.12	0.49	0.45–0.54	< 0.001

*OR* Odds Ratio, *CI* Confidence Interval

Bold values indicate those with a *P-*value < 0.05

### Underweight patients

Relative to normal-weight patients, indication for revision in underweight patients was 131% more likely to be dislocation (OR = 2.31, 95% CI 1.84–2.91, *P* < 0.001), and 63% more likely to be periprosthetic fracture (PPF) (OR = 1.63, 95% CI 1.10–2.41, *P* = 0.02). Cause for revision was 44% less likely to be aseptic loosening (OR = 0.56, 95% CI 0.38–0.84, *P* = 0.01) and 37% less likely to be mechanical complications (OR = 0.63, 95% CI 0.44–0.89, *P* = 0.01).

### Overweight/obese patients

Revisions in patients who were overweight/obese were 19% less likely than normal-weight patients to be due to dislocation (OR = 0.81, 95% CI 0.76–0.87, *P* < 0.001). Cause for revision was 12% more likely to be due to osteolysis/polyethylene wear (OR = 1.12, 95% CI 1.01–1.24, *P* = 0.03), and 10% more likely to be due to periprosthetic joint infection (PJI) (OR = 1.10, 95% CI 1.05–1.15, *P* < 0.001).

### Morbidly obese patients

Revisions in morbidly obese patients were 41% less likely to be due to dislocation (OR = 0.59, 95% CI 0.54–0.65, *P* < 0.001), 8% less likely to be due to mechanical complication (OR = 0.92, 95% CI 0.85–0.99, *P* = 0.02), and 19% less likely to be due to osteolysis/polyethylene wear (OR = 0.81, 95% CI 0.70–0.93, *P* = 0.003). Cause for revision was 90% more likely to be due to PJI (OR = 1.90, 95% CI 1.80–2.00, *P* < 0.001).

## Discussion

BMI has been shown to be associated with a number of adverse outcomes of THA, including complications, readmissions, and revisions [11, 12]. The results of this study add to the existing literature by showing that not only does BMI impact revision risk, but different BMI classes are more likely to undergo rTHA for disparate reasons. Patients who were overweight/obese were less likely to undergo rTHA for mechanical reasons and more likely to undergo rTHA for PJI, whereas patients who were underweight were more likely to undergo rTHA for dislocation or PPF and less likely for mechanical reasons. In other words, although a J- or U-shaped curve may exist with both underweight and overweight/obese patients more likely to undergo rTHA than normal-weight patients, the most likely reasons for these revision procedures differ significantly by BMI class.

The finding that overweight/obese and morbidly-obese patients were more likely than normal-weight patients to undergo rTHA for PJI is in line with existing literature. Numerous studies have shown that obesity is associated with PJI after THA, and this complication is often cited as evidence for introducing BMI cutoffs for performing joint arthroplasties [23, 24]. Evidence indicates that obese patients have a greater risk for infection broadly [25, 26]. This has been postulated due to factors such as malnutrition, hygienic status, and comorbidities [25]. In particular, obese patients often have comorbid diabetes diagnoses, and diabetes has been cited as a risk factor for infection after joint arthroplasties [23, 27]. As the analysis in this study controlled for comorbidity burden using the Elixhauser Comorbidity Index, diabetes as a specific comorbidity was not controlled for, and thus may also partially be a mediating factor in the relationship seen between obesity and the likelihood of PJI being the cause for rTHA. Future work should endeavor to further investigate this association to determine if weight loss, glycemic control, or a combination of both can be used to optimize patients postoperatively to prevent infectious causes for rTHA.

Interestingly, dislocation, mechanical complications, and osteolysis/polyethylene wear were less likely to be the cause of rTHA in morbidly-obese patients compared to normal-weight patients, and dislocation was a less likely cause in overweight/obese patients. This seemingly runs counter to some existing literature, which has shown that obesity is associated with an increase in early revision for dislocation [28, 29]. However, these studies looked specifically at the likelihood of an isolated mechanical complication, rather than in the context of likelihood among all causes for rTHA in morbidly-obese patients. In this study, causes for rTHA were so highly skewed toward infection (51% for morbidly-obese patients vs. 29% for normal-weight patients) that it is important to consider that revision is likely more common overall in obese patients. Therefore, although the predominant cause for revision in these patients may be PJI, surgeons should still emphasize the risk for mechanical complications in patient education and management.

Dislocation was a more likely cause of rTHA in underweight patients compared to normal-weight patients, on the other hand. This is in line with the limited existing literature on underweight THA patients, which has shown an elevated risk of dislocation after THA [17, 30, 31]. This has been postulated to result from a reduced restriction of range of motion due to diminished muscle mass and weakened soft tissue tension around the hip joint [31]. Consequently, in this particular patient population it is important to be particularly vigilant intraoperatively when assessing for dislocation risk, and monitor and educate patients for the potential for dislocation in the course of their recovery. Furthermore, in underweight patients, it may be prudent to consider, where possible, strategies known to reduce risk of dislocation, such as use of dual-mobility constructs or large femoral head sizes, adjustment of implant positioning based on a patient’s spinopelvic relationship, use of technology to ensure appropriate implant positioning, or adjusting surgical approach to a lateral approach to the hip [32].

The results of this study showed that patients who were underweight were more likely to undergo rTHA for PPF. This effect aligns with current understanding of nutrition and biomechanical forces, as low BMI has been shown to be associated with malnutrition and reduced bone mineral density, thus increasing risk for fracture [33, 34]. Furthermore, BMI has been shown to have an inverse relationship with risk of hip fracture broadly, with underweight patients at elevated risk [33]. Understanding that PPF is a more likely cause for revision among underweight patients may guide BMI-specific patient management, with recommendations for nutritional supplementation or BMD-sparing treatments such as bisphosphonates considered more strongly for underweight than normal-weight or overweight/obese patients [35, 36]. Furthermore, additional intraoperative interventions such as cementation of the femoral stem may be considered in underweight patients to reduce the risk of PPF [37, 38].

The results of this study represent a valuable addition to the literature on BMI and rTHA, but several limitations should be noted. This was a retrospective study, and thus, by nature, the results may only be interpreted as association rather than causation. Additionally, as the NIS database was used, there is the potential for error in the fact that data entry is completed by human coders. Moreover, The NIS database only includes patients managed on an inpatient basis, and thus, in more recent years, may not capture a representative sample of THA patients, as THA was removed from the Centers for Medicare and Medicaid Services inpatient-only list in 2020 [39]. However, as the data in this study only included patients through 2020, and revision procedures remain more likely to be managed on an inpatient basis, this should not have significantly impacted the results of our study. The analysis was also limited to the variables available in the NIS database, and thus it could not account for patient-level differences in variables unavailable in the database. Furthermore, the NIS database only presents information on single hospitalizations, so information about the index surgeries for patients, including time from index surgery to revision, could not be included in the analysis. Consequently, for example, it cannot be determined if patients underwent unilateral or bilateral THA for their index procedure, which could have varied by weight status and, in turn, impacted the results. The results of the study for patients who were underweight should also be interpreted with caution, as underweight patients only accounted for 0.4% of the total sample. However, on power analysis of the multivariable logistic regression models, only three statistically insignificant results were found to lack sufficient power (defined as a 1-beta of less than 0.8), specifically underweight vs. normal weight for PJI and overweight/obese as well as morbidly-obese vs. normal weight for arthrofibrosis. Similarly, trends in the results over time could not be investigated, as division of the data across two time periods (2006–2012 and 2013–2020) resulted in too drastic a reduction of the sample size, leading to statistical insignificance due to lack of power and sometimes model breakage in all but the largest causes of revision (PJI and dislocation). Finally, patients were stratified by BMI classes as determined by ICD codes. As studies have shown that ICD codes for BMI are underutilized [19, 40] it is possible that patients in the normal weight control group were actually underweight, overweight, or obese. However, this limitation may be offset by the fact that these ICD codes have a high positive predictive value of a patient’s actual BMI class [19]. Consequently, the results of the analyses in the populations that had BMI codes should still be valid, and may even, in certain cases, be understated.

## Conclusions

In summary, the results of this study demonstrate that patients of the various BMI classes undergo rTHA for different indications. The cause for rTHA among patients who are overweight/obese or morbidly obese is more likely to be due to PJI and less likely to be related to mechanical complications compared to normal-weight patients. On the other hand, the cause for rTHA among patients who are underweight is more likely to be dislocation or periprosthetic fracture and less likely to be mechanical reasons. These differences may relate to the underlying biomechanical and physiological differences seen in patients of each BMI class. Understanding that cause for revision varies among the BMI classes may aid orthopaedic surgeons in developing patient-specific optimization and management protocols to reduce the risk of revision in a more targeted manner.

### Supplementary Information

**Additional file 1: Table S1.** ICD-9 and ICD-10 codes used to identify and classify patients.

**Additional file 2: Table S2.** Definition of quartiles for median household income by patient zip code for the years evaluated.

## Data Availability

The anonymized data collected in the National Inpatient Sample are publicly available for purchase online via the Healthcare Cost and Utilization Project (HCUP): https://hcup-us.ahrq.gov/tech_assist/centdist.jsp.
