# Supplementary material for: The weight of complications: high and low BMI have disparate modes of failure in total hip arthroplasty
Source: Arthroplasty. 2024 Mar 4;6:9. doi: 10.1186/s42836-024-00233-7 (PMC10910669; doi:10.1186/s42836-024-00233-7)
Supplement: Supplementary file 1 — Additional file 1: Table S1. ICD-9 and ICD-10 codes used to identify and classify patients. [file 42836_2024_233_MOESM1_ESM.docx]

**Supplementary Table 1.** ICD-9 and ICD-10 codes used to identify and classify patients.

|  | **ICD-9** | **ICD-10** |
| --- | --- | --- |
| rTHA | 00.70-00.73, 80.05, 81.53, 84.56 and 84.57 | 0SP90XZ, 0SPB0XZ  0SPXXJZ, 0SPXX8Z  0SPA0JZ, 0SPE0JZ  0SRX0XX, 0SUX09Z, 0SWXXJZ, 0SH9X8Z, 0SHBX8Z |
| Underweight (BMI <19 kg/m^2^) | 783.22, V85.0 | R63.6, Z68.1 |
| Overweight/Obese (BMI 25-39.9 kg/m^2^) | 278.00, 278.02, V85.21-V85.25, V85.30-V85.39 | E66.3, Z68.25-Z68.29, E66.01, E66.09, E66.1, E66.8, E66.9, Z68.30-Z68.29 |
| Morbidly Obese (BMI ≥40 kg/m^2^) | 278.01, V85.41-V85.45 | E66.2, Z68.41-Z68.45 |
| Aseptic Loosening | 996.41 | T84.032A, T84.033A |
| Dislocation | 996.42 | T84.022A, T84.023A |
| Mechanical Complications | 996.43, 996.47, 996.49 | T84.012A, T84.013A, T84.092A, T84.093A |
| Periprosthetic/Total Joint Fracture | 996.44 | M97.11XA, M97.12XA |
| Osteolysis/Polyethylene Wear | 996.45, 996.46 | T84.052A, T84.053A, T84.062A, T84.063A |
| Periprosthetic Joint Infection | 996.60, 996.66, 996.67, 996.69 | T84.50XA, T84.53XA, T84.54XA, T84.60XA, T84.7XXA, T85.79XA |
| Arthrofibrosis | 718.46, 718.56 | M24.561-M24.569, M24.661-M24.669 |
| Other Complications | 996.40, 996.77, 996.78, 718.86 | T84.498A, T84.81XA-T84.89XA, T84.9XXA, M23.50-M23.52, M23.8X1-M23.8X9 |
