# Supplementary material for: The weight of complications: high and low BMI have disparate modes of failure in total hip arthroplasty
Source: Arthroplasty. 2024 Mar 4;6:9. doi: 10.1186/s42836-024-00233-7 (PMC10910669; doi:10.1186/s42836-024-00233-7)
Supplement: Supplementary file 2 — Additional file 2: Table S2. Definition of quartiles for median household income by patient zip code for the years evaluated. [file 42836_2024_233_MOESM2_ESM.docx]

**Supplementary Table 2.** Definition of quartiles for median household income by patient zip code for the years evaluated.

| Year | Q1 | Q2 | Q3 | Q4 |
| --- | --- | --- | --- | --- |
| 2006 | 1 - 37,999 | 38,000 - 46,999 | 47,000 - 61,999 | 62,000+ |
| 2007 | 1 - 38,999 | 39,000 - 47,999 | 48,000 - 62,999 | 63,000+ |
| 2008 | 1 - 38,999 | 39,000 - 48,999 | 49,000 - 63,999 | 64,000+ |
| 2009 | 1 - 39,999 | 40,000 - 49,999 | 50,000 - 65,999 | 66,000+ |
| 2010 | 1 - 40,999 | 41,000 - 50,999 | 51,000 - 66,999 | 67,000+ |
| 2011 | 1 - 38,999 | 39,000 - 47,999 | 48,000 - 63,999 | 64,000+ |
| 2012 | 1 - 38,999 | 39,000 - 47,999 | 48,000 - 62,999 | 63,000+ |
| 2013 | 1 - 37,999 | 38,000 - 47,999 | 48,000 - 63,999 | 64,000+ |
| 2014 | 1 - 39,999 | 40,000 - 50,999 | 51,000 - 65,999 | 66,000+ |
| 2015 | 1 - 41,999 | 42,000 – 51,999 | 52,000 – 67,999 | 68,000+ |
| 2016 | 1 – 42,999 | 43,000 – 53,999 | 54,000 – 70,999 | 71,000+ |
| 2017 | 1 – 43,999 | 44,000 – 55,999 | 56,000 – 73,999 | 74,000+ |
| 2018 | 1 – 45,999 | 46,000 – 58,999 | 59,000 – 78,999 | 79,000+ |
| 2019 | 1 – 47,999 | 48,000 – 60,999 | 61,000 – 81,999 | 82,000+ |
| 2020 | 1 – 49,999 | 50,000 – 64,999 | 65,000 – 85,999 | 86,000+ |

Definition of quartile ranges taken from NIS description of data elements
